# Marine Bacterial Dextranases: Fundamentals and Applications

**DOI:** 10.3390/molecules27175533

**Published:** 2022-08-28

**Authors:** Noora Barzkar, Olga Babich, Rakesh Das, Stanislav Sukhikh, Saeid Tamadoni Jahromi, Muhammad Sohail

**Affiliations:** 1 Department of Marine Biology, Faculty of Marine Science and Technology, University of Hormozgan, Bandar Abbas 74576, Iran; 2 Institute of Living Systems, Immanuel Kant Baltic Federal University, 236016 Kaliningrad, Russia; 3 Department of Paraclinical Sciences, Faculty of Veterinary Medicine, Norwegian University of Life Sciences (NMBU), 1432 Ås, Norway; 4 Persian Gulf and Oman Sea Ecology Research Center, Iranian Fisheries Sciences Research Institute, Agricultural Research Education and Extension Organization (AREEO), Bandar Abbas 14578, Iran; 5 Department of Microbiology, University of Karachi, Karachi 75270, Pakistan

**Keywords:** dextran, dextranase, marine bacteria, biochemical properties, dental plaque

## Abstract

Dextran, a renewable hydrophilic polysaccharide, is nontoxic, highly stable but intrinsically biodegradable. The α-1, 6 glycosidic bonds in dextran are attacked by dextranase (E.C. 3.2.1.11) which is an inducible enzyme. Dextranase finds many applications such as, in sugar industry, in the production of human plasma substitutes, and for the treatment and prevention of dental plaque. Currently, dextranases are obtained from terrestrial fungi which have longer duration for production but not very tolerant to environmental conditions and have safety concerns. Marine bacteria have been proposed as an alternative source of these enzymes and can provide prospects to overcome these issues. Indeed, marine bacterial dextranases are reportedly more effective and suitable for dental caries prevention and treatment. Here, we focused on properties of dextran, properties of dextran—hydrolyzing enzymes, particularly from marine sources and the biochemical features of these enzymes. Lastly the potential use of these marine bacterial dextranase to remove dental plaque has been discussed. The review covers dextranase-producing bacteria isolated from shrimp, fish, algae, sea slit, and sea water, as well as from macro- and micro fungi and other microorganisms. It is common knowledge that dextranase is used in the sugar industry; produced as a result of hydrolysis by dextranase and have prebiotic properties which influence the consistency and texture of food products. In medicine, dextranases are used to make blood substitutes. In addition, dextranase is used to produce low molecular weight dextran and cytotoxic dextran. Furthermore, dextranase is used to enhance antibiotic activity in endocarditis. It has been established that dextranase from marine bacteria is the most preferable for removing plaque, as it has a high enzymatic activity. This study lays the groundwork for the future design and development of different oral care products, based on enzymes derived from marine bacteria.

## 1. Introduction

**Figure 1:**
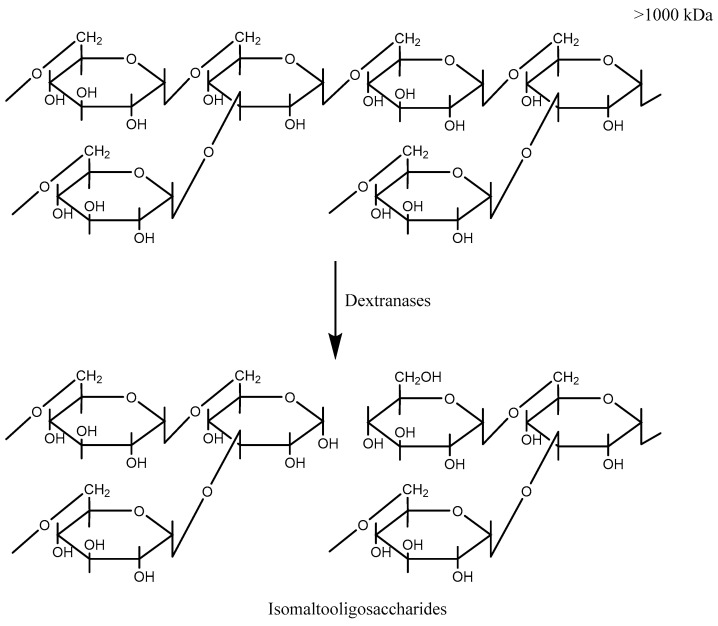
Cleavage of dextran by dextranase.

Dextran is a natural polymer (Figure 1) with a main chain of molecules linked by α-1,6 bond, and side branches linked by α-1,3 bonds. Dextran is renewable and biodegradable chemical resource with the desirable properties of nontoxicity, stability and water solubility [1]. Chemically modified dextran can act as a potential drug carrier, as it is degraded by dextranase and releases the drug of choice at the target site [2,3].

Dextranases (α-1,6-D-glucan-6-glucanohydrolase; EC 3.2.1.11) randomly hydrolyze α-1,6-linkages of dextran and release isomaltooligosaccharides (IGs) of various sizes [4]. It is extremely rare that the product of hydrolysis is glucose. Large dextran compounds break down more easily than small ones due to the increased contact of dextran with dextranase molecules. IGs are applied in many processes including in sugar industry, in medicine making, and in dentistry. Sugar industries employ dextranase to degrade any residual dextran in sugar juice, which in turn reduces the viscosity of the juice and improves the yield of sucrose [5,6,7]. In medicine, dextranases are used to convert high molecular weight dextran into low molecular weight compounds; the dextran of specific molecular weight has an antithrombotic effect and hence can substitute blood under emergent conditions [8,9]. In dentistry, dextranases are applied to degrade dental plaque glucans and to prevent glucan formation [10]. Lately, the attention towards dextranases increased due to its recent advancement in prebiotics synthesis [11].

Active centers (Figure 1) are α-1,6, α-1,3 and α-1,4 bonds [12].

The effectiveness of dextranase depends on the acidity, solids content, temperature, contact time, agitation, dextran concentration and the source, activity and dosage of the dextranase used. The optimal acidity range for dextranase activity is 5.0–6.0. Moreover, lower values in this range are preferred. In addition, the effectiveness of dextranase depends on the temperature of the medium.

Historically, dextranase was discovered in *Cellvibrio fulva* around 80 years ago [13]. Currently various dextranase-producing microorganisms have been described including bacteria, filamentous fungus and yeasts [9,12,14]. The bacterial genera, *Bacillus* [15,16,17,18], *Shewanella* [19], *Thermoanaerobacter* [20], *Arthrobacter* [21], *Cytophaga* [22], *Paenibacillus* [23,24,25] and *Streptococcus* [26] have been reported to produce dextranases in good yield. Among fungal species, *Paecilomyces lilacinus* [27] and the species of *Penicillium* [28,29], *Hypocrea* [30], *Pochonia* [31], *Chaetomium* [32], *Talaromyces* [3], and *Aspergillus* [33] are reportedly promising producers of dextranases. The microbial sources of dextranases from marine environment have also been described, from bacteria [34,35,36,37] and from fungi [38]. The concerns raised by the US Food and Drug Administration (FDA) over the use of fungal dextranase [39,40] have diverted the attention of researchers to explore alternative or new sources of dextranase.

The marine environment is considered as a source of novel microorganisms with particular trait of cold-adaptation and salt and alkali tolerance can serve as a unique reservoir for dextranase [41]. Cold-adapted (cold-active) enzymes are already in demand in various commercial sections, particularly for formulation of improved detergents, for the development of environmental biosensors, and as a carrier matrix for bioremediation [42]. Moreover, cold-adapted dextranases are also uniquely suited for the synthesis of novel medicines. Likewise, marine alkaline dextranases are compatible with alkaline tooth-rinse products in the treatment of oral dental plaques [43]. Indeed, the attributes of high salt tolerance, thermostability, and low optimum temperature of marine dextranases render them suited for the human oral environment.

Marine bacterial dextranases have also exhibited their ability to prevent biofilm formation by *Streptococcus mutans*. Therefore, the prospects of utilizing more marine bacterial dextranases for the development of oral care products are quite considerable and envisage further research in this direction.

In addition to their applications in research, dextranases are also applied to prepare dextran and its derivative. The enzyme is also applied in molasses and beverage processing in food industries [44].

It is used to study the structure of other polysaccharides and to obtain oligosaccharides. Dextranase-cleaved polysaccharides are used in the cosmetic and medical industries as cryoprotectants, and in the food industry as stabilizers. Medical sterile blood substitutes (dextran of a certain molecular weight) are also produced using dextranase. The newest use of dextranase is for labeling in cancer therapy. In the sugar industry, this enzyme is used to remove sucrose sludge [44,45,46,47].

This research aimed to investigate the classification and properties of dextran hydrolyzing enzymes, as well as the methods for obtaining, biological properties, and practical applications of marine dextranases. This study compared dextranases from marine bacteria with dextranases from other microorganisms along with the effect of marine dextranase on plaque removal is described. Dextranases have a wide spectrum of bactericidal and bacteriostatic activity. Dextranases are classified based on the types of catalyzed reactions and the specificity of the resulting product. This review precisely presents dextranase-producing bacteria isolated from various marine sources: shrimp, fish, seaweed, sea silt and sea water and other alternative sources of dextranases (macro- and microfungi, bacteria).

The novelty of this study is that it is the first time that dextranases produced by alternative sources like marine bacteria have been analyzed, dextranases produced by micro- and macrofungi were compared, and the advantages and disadvantages of various dextranases have been discussed. In addition, the impact of marine dextranases on plaque removal has been summarized for the first time. This information will be used to design and develop oral care (biofilm removal) products using products and enzymes derived from marine bacteria.

## 2. Classification and Properties of Dextran—Hydrolyzing Enzymes

The search for organisms that produce a large amount of the enzyme needed to degrade dextran began in the 19th century. Organisms producing trace amounts of both intracellular and extracellular dextranase were isolated in the late 1940s, but it was not until the 1950s that Penicillium species producing large amounts of extracellular dextranase were reported.

In the early 1970s, the first attempts were made to purify and characterize *Penicillium dextranase*. In the late 1970s, research began on the use of dextranase for the treatment of caries [48].

In the 1990s, the *Penicillium dextranase* gene was cloned and expressed from Pichia pastoris. An early classification system for dextranase and other dextran hydrolyzing enzymes was also developed using sequence-analysis software (GCG, Version 8.0.1, Madison, WI, USA) [49].

The crystal structure of *Penicillium minioluteum* was revealed by Larsson et al. in 2003 [50]. In this study, it was suggested that the reaction mechanism proceeds by pure inversion (rather than retention) of the anomeric carbon.

Extracellular dextranase is encoded by the dex gene. Post-translational modification includes signal peptide cleavage and N-glycosylation.

Dextranase enzymes belongs to two families of glycoside hydrolases, either 49 or 66, which do not share significant sequence similarity. *Penicillium* and *Arthrobacter dextranases* belong to family 49, while *Streptococcus* dextranases belong to family 66 [4].

*Penicillium dectranase* and related enzymes contain two domains. The first domain resembles the folding of an immunoglobulin and consists of 200 amino acid residues forming 13 β-chains. The β-sandwich is formed by nine of these strands, with all strands except 5 and 13 being antiparallel. The second domain contains a right-sided parallel β-helical fold containing 3 parallel β-sheets [50]. These two domains are connected by a large interface that contains 29 amino acids, completely conserved in the family of glycoside hydrolases 49. Disulfide bridges are formed by four of the six cysteines present in the protein, none of which is conserved in the family of glycoside hydrolases 49 [50].

Dextran-degrading enzymes belong to different groups of carbohydrases and transferases. Based on their mode of action, these can be categorized as endo- and exo-dextranases [51,52]. There are known endodextranases isolated from molds *Penicillium luteum* ATCC 9644, *Penicillium funiculosum*, *Penicillium funiculosum* NRRL, *Penicillium lilacinum* NRRL, *Penicillium notatum*, *Penicillium acualeatum*, *Aspergillus carneus*, *Chatomium gracile*, *Fusarium* sp., *Sporotrix schencki*, which have extracellular localization. There are also known endodextranases isolated from the yeast *Lipomyces starkeyi* ATCC 20825, *Lipomyces starkeyi* KSM 22, *Lipomyces starkeyi* IGC 4047. Endodextranases are also obtained from bacteria *Bacteroides oralis* Ig4a, *Flavobacterium* sp. M-73, *Pseudomonas* sp. All of them have extracellular localization [4].

There are known exodextranases from bacteria *Bacteroides oralis* IG 4a, *Arthrobacter globiforms* I-42, *Pseudomonas* sp., *Streptococcus mitis* ATCC 903, from yeast *Lipomyces lipofer* IGC 4042. All exodextranases are extracellular, except for exodextranases derived from *Pseudomonas* spp. and *Streptococcus mitis* ATCC 903. These exodextranases are intracellular [4].

Furthermore, the Nomenclature Committee of the International Union of Biochemistry and Molecular Biology (IUBMB) considers the types of reactions catalyzed by dextranases and the product specificity to classify these enzymes as simply dextranase (EC3.2.1.11), glucan-1,6-α-D-glucosidases (EC3.2.1.70), glucan-1,6-α-isomaltosidases (EC3.2.1.94), dextran 1,6-α-isomaltotriosidases (EC3.2.1.95), and branched-dextran exo-1,2-α-glucosidases (EC3.2.1.115) [53]. The reaction catalyzed by Cycloisomaltooligosaccharide glucanotransferase (CITase) also yields hydrolyzed dextran as a byproduct (Table 1) [54]. Interestingly, α-glucosidase (EC3.2.1.20) that is considered as an unrelated enzyme to dextran synthesis also catalyzes the reaction similar to those of exo dextranases (EC3.2.1.70) [55]. Based on the similarities in the amino acid sequences, glycosylhydrolases and glycosyltransferases have been divided into different families [56,57] (http://www.cazy.org, accessed on 1 July 2021) in the Carbohydrate Active Enzymes (CAZy) database. CAZy database was developed with the aim to classify the enzyme families with structurally related domains for catalysis of glycosidic bonds and for carbohydrate-binding (functional domains). An analogous classification system for dextran-hydrolyzing enzymes has also been proposed to divide the enzymes into four families [58] but the system did not gain much attention and it’s details are beyond the scope of this review. In addition, CAZy database has been developed to integrate structural properties of the enzymes with its mechanical features; therefore, the enzymes with different substrate specificities have been placed in the same family, while the enzymes with the ability to catalyze the same substrate are sometimes placed in different families [59]. In this database, dextran-glucosidases (EC3.2.1.70) have been included in glycosylhydrolase families 13 and 15 (http://www.cazy.org, accessed on 1 July 2021). The families 27 and 49 contains structurally different enzymes of isomaltodextranase (EC3.2.1.94) and isomaltotriosidase (EC3.2.1.95). While the families 49 and 66 contain endodextranases, the two families, however, do not harbour similarities in the sequences. Moreover, CITase from *Bacillus circulans* is classified in family glycosylhydrolase 66 and shares a high homology with endodextranases. The enzyme (α-1,6-D-glucan 6-glucanohydrolase; EC 3.2.1.11) that cleave dextran chain at α-1,6 glucosidic bond and release oligosaccharides are included in the GH families 49 and 66 based on amino acid sequence homology [4].

**Figure 2:**
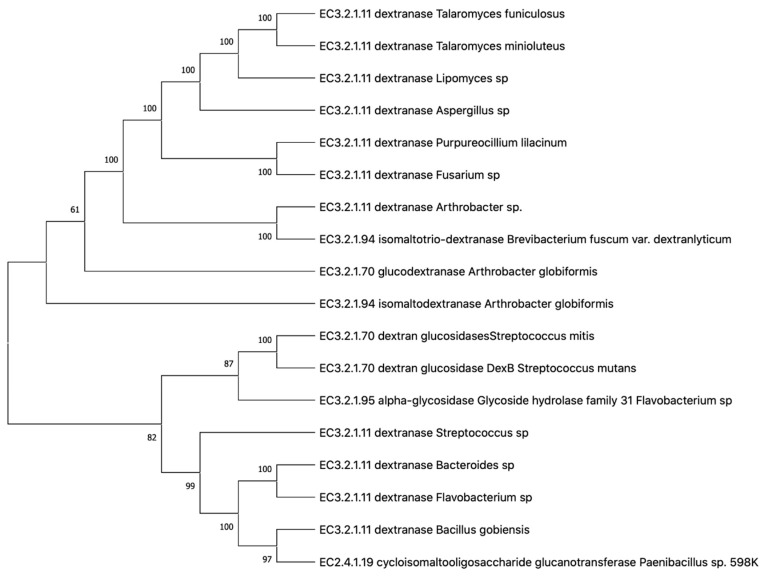
Phylogenetic tree of dextranases from various sources. Phylogenetic tree performed with Neighbor-Joining method by MEGA X of various sources of dextranases, including those identified from bacteria, Fungi etc. The tree was drawn to scale, with branch lengths in the same units as those of the evolutionary distances used to infer the phylogenetic tree. The percentage of replicate trees in which the associated taxa clustered together in the bootstrap test (10,000 replicates) are shown next to the branches.

Figure 2 demonstrates the phylogenetic tree of dextranases from various sources.

## 3. Marine Dextranase

Oceans are the largest ecosystem and make this planet ‘blue’ by covering almost two third of the planet’s surface, and they are the most ancient habitat of living organisms [60]. The difficulties in sampling impede complete exploration of the marine organisms; therefore, it is believed that the habitat can still provide new novel species [61,62,63]. Salinity and generally cold and alkaline environments are peculiar features of oceans that influence the life in that habitat which is known for harboring unique catalysts [64,65,66,67,68,69,70,71,72,73,74,75]. Therefore, potential applications of marine-derived catalysts can easily be conceived. Although not enough research has been devoted to the study of marine dextranases, yet there are few reports on *Catenovulum* sp. [76,77,78,79], *Arthrobacter* sp. [36,37,80,81,82,83], *Cellulosimicrobium* sp. [35], and *Bacillus* sp. [84] and marine fungi, *Aspergillus* sp. [38] indicate about dextranase production potential of marine microorganisms. Interestingly, marine yeasts have not been reported for the production dextranases.

Marine ecosystems in China, such as Gaogong island seacoast in Jiangsu [77,79], the lower part of the intertidal zone of Lianyungang port [34], and Haizhou Bay in Jiangsu a [76,84] have been explored for the isolation of dextranase producing marine bacteria. Marine bacterial dextran producers have been isolated from shrimps [85], sea water [76,79], beach mud, fishes, and seaweeds [34], and sea mud, seaweed, and seawater [76].

Moreover, dextranase from marine bacteria has also been subjected to purification and characterization (Table 1). For that purpose, routine protein purification protocols, employing concentration by using methods (NH_4_)_2_SO_4_ precipitation [83] or combination of Alcohol and (NH_4_)_2_SO_4_ precipitation [77] or ultrafiltration [34,76,79,84] were carried out and the concentrated fractions were subjected to different chromatographic techniques. In the Table 1, a list of the purification strategies used for purifying these dextranases is depicted. Table 1 represents dextranases from microorganisms that use Dextran T500 as a nutrient medium which hydrolyze α-1.6 bonds [29]. 

**Table 1:** Dextranase producing bacteria isolated from different marine sources and their isolation and purification details.

Species	Enzyme	Isolated from	Concentration Method	Purification Method	Mw (kDa)	Purification (-Fold)	Specific Activity (U/mg)	Ref
*Arthrobacter* sp.	Dex410	Beach mud, fishes, and seaweeds	Ultrafilteration	DEAE-Sepharose	64	-	11.9	[34]
*Catenovulum agarivorans* MNH15	-	Sea mud, seaweed, and seawater	Ultrafilteration	-	110	-	-	[76]
*Catenovulum* sp. DP03	Cadex	Sea water	Alcohol and (NH_4_)_2_SO_4_ precipitate	Ion exchange chromatography	75	29.6	2309	[77]
*Catenovulum* sp. DP03	Cadex2870	Sea water	Ultrafilteration	Ni-NTA resin		29.9	46.3	[79]
*Arthrobacter oxydans* KQ11	-	-	(NH_4_)_2_SO_4_ precipitate	Ion-exchange chromatography on Q Sepharose Fast Flow	66.2	43.00	36.38	[83]
*Bacillus aquimaris* S5	BaDex	Shrimps caught	Ultrafilteration	Magnetic bead (His-tag protein purification beads)	70	-	-	[84]

For the purification of dextranases, Ren et al. [74] used the following methods in comparison: ultrafiltration, ethanol precipitation, ammonium sulfate precipitation, thin layer and ion exchange chromatography [34]. The crude Cadex dextranase activity was 77.9 U/mg, and after successive purification by ultrafiltration, ethanol precipitation, and ammonium sulfate precipitation, 163.5 U/mg, 223.3 U/mg, and 341.6 U/mg, respectively. When ion exchange chromatography was used for purification, Cadex was purified 29.6 times with a specific activity of 2309 U/mg of protein and a yield of 16.9%. After ultrafiltration, about 5.4% of enzymatic activity was lost, and the specific activity of the enzyme increased by 48.7%. Ethanol precipitation and ammonium sulfate precipitation were used to remove polysaccharides and proteins [34]. A study [86] purified and characterized dextranase from *Arthrobacter oxydans* G6-4B. In addition, using anion exchange chromatography, dextranase was successfully purified by 32.25 times with a specific activity of 288.62 U/mg of protein and a molecular weight of 71.12 kDa.

In addition, the physicochemical properties of marine bacterial dextranases were presented (Table 2). 

Generally, mold fungi dextranases have optimal pH 4.5–5.5, and temperature 50–60 °C; yeast dextranases have optimal pH 5.0 and temperature 55 °C; bacteria dextranases have optimal pH 5.0–7.5, and temperature 40–60 °C.

Dextranase (Dex410) from marine *Arthrobacter* sp. was isolated and described in a study [34]. Dex410 was a 64 kDa endoglycosidase. The specific activity of Dex410 was 11.9 U/mg at optimal pH values of 5.5 and 45 °C. The main end products of Dex410 were isomaltotriose, isomaltotheraosis, and isomaltopentaosis from dextran hydrolysis [34]. High thermal stability is one of the conditions for the use of dextranase. Previous results [76,87] have shown that the optimum temperature for the dextranase reaction is generally in the range of 25 °C to 60 °C, possibly related to the physiological characteristics and survival conditions of the various strains. Generally, the rate of a chemical reaction increases with increasing temperature. However, at high temperatures, the enzyme protein, like all proteins, becomes irreversibly deformed, causing the enzyme to lose its catalytic activity. Similarly, within a certain pH range, the enzyme exhibits catalytic activity, but outside the pH range, the activity of the enzyme is weakened or even lost. Deng. et al. [79] reported that expression of marine bacterial dextranase in *E. coli* was inactive at 50 °C for 1 h. The pH values of the medium were varied from acidic (pH 5.5–6.0) to neutral or slightly alkaline (pH 7.0–8.0) in a relatively wide range. At the stage of purification of dextranase, the temperature usually reached 65 °C and above. Compared to dextranase from most marine bacterial strains, dextranase from *A. oxydans* G6-4B significantly improved heat tolerance; such characteristics were more suitable for the application of high temperature dextran hydrolysis processes. Nevertheless, the pH and stability of metal ions were unsatisfactory [88,89]. Relatively high activity of the enzyme could be maintained only in a medium from neutral to slightly alkaline, in contrast to dextranase from *Streptomyces* sp. NK458 and *Chaetomium gracile* [90], which showed high enzymatic activity in the pH range of 5.0 to 10.0 (>50% activity). Thus, studies on thermal and acid stability need to be further continued and improved in order to fully utilize dextranase from marine bacteria in industry.

Dextranase from G6-4B exhibited relatively higher activity at 40–60 °C. A temperature of 55 °C was the optimal temperature for further experiments. At temperatures above 70 °C, almost 66% of enzyme activity (190.49 U/mg) was lost compared to the optimum temperature. The thermostability chart showed that dextranase activity remained at least 93% (268.42 U/mg) for 3 h at 50 °C and almost 60% (173.17 U/mg) was maintained after storage at 60 °C for 5 h. It was shown that the highest activity was at pH 7.5, and a relatively high level of activity remained in the pH range of 7.0–9.0, which is consistent with an increase in the pH of the medium above 7.0. However, under acidic conditions (at pH 3.0–6.0), more than 50% of the enzyme activity was lost. In general, dextranases from strain G6-4B can maintain a relatively high level of activity in a neutral or slightly alkaline environment. The optimal reaction conditions were 55 °C and pH 7.5, and it remained relatively stable in the pH range of 7.0–9.0 and below 60 °C, while being significantly inhibited by metal ions such as Ni ^+^, Cu ^2+^, Zn ^2+^, Fe ^3+^, and Co ^2+^. Notably, unlike in previous studies, the dextran hydrolysates were mostly isomaltoiose (more than 73%), with no glucose, and the hydrolysates were relatively stable after 30 min; dextranase activity had a large effect on the hydrolyzate [86]. 

The ionization of the enzyme is the primary reason for the influence of the medium pH on enzymatic activity. A change in the environment induces a change in the degree of dissociation and protonation of various chemical groups of the enzyme protein molecule, as well as its total charge, resulting in a change in the polypeptide chain’s conformation. This affects the ability of the enzyme to attach the substrate. This process is strongly influenced by the change in the charge of chemical groups located in the active center of the enzyme [85]. The most long-term use of dextranases is observed at stable temperature and pH parameters [84,85]. Table 2 includes dextranases from marine bacteria the activity of which is influenced by metal ions. There are certain metal ions that affect dextranase activity, such as Fe ^2+^, Li ^+^ [91]. For instance, the presence of Co ^2+^, Mn ^2+^, Ca ^2+^ accelerated the activity of dextranase from *A. allahabadii* X26 [91].

Lai et al. [76] studied the effect of metal ions on dextranase activity from marine bacterial strain MNH15, *Catenovulum agarivorans*. The presence of Sr^2+^ had a positive effect on dextranase (Cadex) activity, it was enhanced to 128.71%, while Ni^2+^, Cd^2+^, Fe^3+^, Li^+^, Cu^2+^, and Co^2+^ had a strong inhibitory effect [76]. Interestingly, when dextranase from another species (DP03) of the same genus, *Catenovulum*, was investigated, very little effect of SrCl_2_ was observed (Table 3) indicating about the structural diversity in the enzymes from closely related species. In addition to that, positive impact of Mn^2+^ and inhibitory effect of Cu^2+^, Fe^3+^, Zn^2+^, Cd^2+^, Ni^2+^, and Co^2+^ was also described [77]. In a study by Wang et al. [81], the positive effect of Ca^2+^ and negative influence of Ni^2+^ and Fe^3+^ on dextranase activity was reported. Additionally, the presence of 10 mM Co^2+^, 0.02% xylitol and 1% alcohol enhanced dextranase activity by 196%, 132.25%, and 110.37%, respectively [83] (Table 2).

In addition, there are few recombinant dextranases from marine sources were investigated by selected anonymous authors for their characterization and possible applications. Table 3 represents dextranases from marine bacteria that heterologically converted dextran to the desired functional product with desired plaque-breaking properties.

The researchers at the Medical School of the University of Florida heterologously expressed dextranase from *Streptococcus salivaris* in *Escherichia coli* [85] that was followed by many similar studies. Japanese researchers adopted a different strategy to express dextranase from *Arthrobacter* sp. CB-8 in an oral bacterium, *Streptococcus gordinii*, to observe any effect in the prevention of dental caries [85]. 

Dong et al. [84] studied dextranase BaDex from the marine bacterium *Bacillus aquimaris* S5. The BaDex gene was 1788 bp long and encoded 573 amino acids. Using bioinformatics to predict and analyze the amino acid sequence of BaDex, it was found that the isoelectric point and the coefficient of instability were 4.55 and 29.22, respectively. The average hydrophilicity was 0.662 [84].

A study [79] cloned and expressed cold-adapted dextranase from the marine bacteria *Catenovulum* sp. DP03. Recombinant dextranase (Cadex2870) contained an intact open read length of 2511 bp and encoded 836 amino acids. The expression condition for the recombinant strain was 0.1 mM isopropylthiogalactoside and the reduced temperature was 16 °C [79].

## 4. Comparison of Dextranases from Marine Bacteria with Those from Other Sources of Microorganisms

Dextranase is found in plants, mammalian tissues, fungi, including yeast, and bacteria; amongst them fungi are a rich source of this enzyme [87]. Dextranase is used in many industries, but it is of limited use due to its low stability in aggressive environments, which negatively affects dextranase activity [76]. There are numerous causes of dextranase instability in oral care products, including temperature, acidity, alkalinity, surfactants, and other oral care product components [92]. Fungal dextranase is widely used in the food industry as it is safe and easy to obtain after harvesting mushrooms [93].

The study [94] compared the effect of extracts from various fungi (*Lentinula edodes* (shiitake), *Pleurotus eryngii*, *Hypsizigus marmoreus*, and *Pholiota nameko*) on the biofilm resistance of *S. mutans* XC and *S. sobrinus* ATCC 33485. It was found that extracts of shiitake, *P. eryngii*, and *H. marmoreus* could reduce biofilms formed by *S. sobrinus* only when using high concentrations of dextranases contained in extracts of the mycothallus of fungi. In addition, after the action of fungal extracts on biofilms ceased, *S. sobrinus* biofilms quickly recovered [94].

The study [95] demonstrated that when *Penicillium* sp. dextranase is used, activity-dependent degradation of higher oligosaccharides can be observed. Therefore, the isomaltose units are released from the non-reducing ends of the higher oligosaccharides. Interestingly, the higher dextranase activity of the *Chaetomium* sp. fungus also led to some further hydrolysis of the biofilm (although linear oligosaccharides are usually completely hydrolyzed at this level of activity). As it can be seen from the chromatograms, this compound (and most likely other branched oligosaccharides eluting later) was degraded to oligosaccharides. However, active *Chaetomium* sp. dextranase can remove isomaltosyl units from the non-reducing end of branched oligosaccharides. It is noteworthy that the proportion of oligosaccharides also increased with increasing enzyme activity. Most likely, this is the result of the hydrolysis of higher oligosaccharides with dimeric side chains.

The two bacterial dextranases showed distinctly different product characteristics compared to the dextranases from *Chaetomium* sp. and *Penicillium* sp. It is worth noting that oligosaccharides (O 6-linked glucose unit) were not detected in significant amounts in hydrolysates obtained with both bacterial dextranases, indicating that they (unlike fungal dextranases) can cleave the 1,6-bond in the 1,4, 6-linked glucose unit. Thus, bacterial dextranases showed a clearly higher activity against branched chain dextrans than fungal enzymes, as also indicated by the usually high intensity of the peaks and the high content of linear isomaltooligosaccharides [95]. Furthermore, some bacterial producers have the ability to simultaneously biosynthesize several α-glucosidases, which differ in localization and mechanism of action.

Table 4 compares the characteristics of sources for producing dextranase based on the results of literature source analysis [16,96,97].

Thus, based on the table data, it can be concluded that dextranase from marine bacteria is the most preferable to use and has a high enzymatic activity.

However, it was noted that dextranase should be chosen carefully, particularly for each case, and for structural analysis or technological purposes [95]. Optimal conditions for the use of plant, fungal, and bacterial dextranases, depending on the application, can be used to selectively produce high yields of certain oligosaccharides.

Studies [98] demonstrated the diversity of structures of dextranases of various origins. With regard to incubation conditions, high activity of all enzymes at about 40 °C and slightly acidic pH are in most cases the preferred growth conditions for the initial dextranase-producing microorganisms. Hydrolysis of linear dextrans showed that dextranases from *B. thetaiotaomicron*, *S. salivarius*, and *Chaetomium* sp., which have different structures, ultimately lead to the formation of isomaltose. However, isomaltose and isomaltotetraose have been found as end products of hydrolysis by dextranase from *Penicillium* sp. This unique spectrum of products is the result of a disproportionation reaction in which isomaltothriose is converted to isomaltose and isomaltotetraose. For O 3 -branched dextrans, the product patterns obtained using fungal and bacterial dextranases of different structures were comparable within the two groups but differed between them. Namely, dextranases from *Chaetomium* sp. and *Penicillium* sp. were able to hydrolyze O 3-branched dextrans to a greater extent. However, the same conclusions about the structures of dextranases can be drawn from the profiles of oligosaccharides. In addition, bacterial dextranases also hydrolyzed O.4-branched dextrans to a lesser extent than fungal dextranases, resulting in higher proportions of longer branched oligosaccharides. Notably, dextranase from *Penicillium* sp. was able to hydrolyze this type of dextran to a clearly higher degree than the dextranase from *Chaetomium* sp. Therefore, the structural analysis of the enzyme, which is necessary for technological purposes, is of great importance. However, differences between enzyme structures can also be used to selectively obtain certain oligosaccharides in higher yields [98].

In a study [99], the crystal structure of the non-liganded dextranase *Flavobacterium johnsoniae* (FjDex31 A) was determined with a resolution of 2.0 Å. The crystal belongs to space group P1 and four molecules, Mol-A, Mol-B, Mol-C and Mol-D, are present in the asymmetric unit. The structures of the four molecules are almost identical and show a continuous electron density for almost all amino acid residues from Glu25 to Asn836. Notably, the Leu308-Gln309 residue pair adopts the cis configuration. FjDex31A consists of four domains: an N-terminal β-sandwich domain (residues 25–242), a catalytic domain (243–600), a proximal C-terminal β-sandwich domain (601–681), and a distal C-sandwich domain and terminal β-sandwich domain (682–836). The catalytic domain is located at the end of the third and at the end of the fourth β-chain.

The search for structural similarity was carried out with Cellvibrio japonicus (CjAgd31B). CjAgd31B carries one glucosyl residue from α-1,4-glucans in transglucosylation reactions. FjDex31A is also highly homologous to Listeria monocytogenes GH31 enzymes 3-α-isomaltosyltransferase, which catalyzes transglycosylation to form α-1,3 bonds in cycloalternane (*L. monocytogenes* cycloalternane-forming enzyme and Blautia obeum α-glucosidase, which efficiently hydrolyzes α-1,6 bond of isomaltose. FjDex31A is also homologous to some GH31 enzymes, such as *E. coli* α-xylosidase and *Pseudopedobacter saltans* α-galactosidase [99].

## 5. Effect of Marine Dextranase on Dental Plaque Removal

The effect of fluorine is on the process of enamel dissolution of particular interest. Penetrating into the crystal lattice, it contributes to the formation of hydroxyfluoropapatite, a compound that is characterized by greater acid resistance and lower solubility [100]. In biopsy specimens of the enamel of permanent teeth with fluorosis, a decrease in calcium was noted with a normal content of phosphorus [101].

One of the main properties that determine the resistance of enamel is microhardness. Previous studies have determined the microhardness of hard tissues of animal and human teeth in normal conditions and in various pathological processes [102,103], in thyroid pathology [104,105], the influence of various biogeochemical factors [106] and prophylactic agents on the level of microhardness [107,108,109].

There are a large number of microorganisms in the oral cavity of animals [110]. Dental plaque biofilm is composed of many microbial floras that adheres to the surface of teeth, which has an important impact on oral health. Many oral diseases are related to the formation of dental plaque [111,112,113]. Current knowledge suggests that *Streptococcus mutans* plays a significant role in the forming of dental caries. Therefore, *S. mutans* is often used as a model strain in research in constructing dental plaque biofilms [114]. The formation of dental plaque biofilm changes dynamically with time. Kolenbrander [115] found that the initial stage of dental plaque biofilm is mainly carried out by *Streptococcus*, while in the later stage *Bacillus* spp., Filamentous bacteria and Actinomycetes play important roles. *S. mutans*, *Streptococcus sobrinus* and *Streptococcus sanguis* are the early strains of dental plaque biofilm formation and play a key role in the formation of dental caries [114,116]. Dextranase has represented excellent effect value in the prevention and removal of dental plaque biofilm [114]; therefore, many workers have exploited dextranase to remove and defend dental plaque biofilm. In 1968, Fitzgerald et al. for the first-time proposed dextranase as a therapeutic agent for dental caries. In vitro experiments supported their hypothesis as the hydrolysis of α-1,6-glycosidic bonds in dental plaque by dextranase was evident [117]. Later on the experiments in animal model confirmed that dextranase could effectively inhibit hamster’s dental caries [118].

Ren and colleagues [77] designed *Arthrobacter oxydans* KQ11 dextranase containing mouthwash and evaluated the efficacy of these mouth washes against biofilm formation by *Streptococcus mutans* using Response Surface methodology approach. The study provided the optimal levels of the factors as 2.16 g L^−1^ ZnSO_4_, 14 g L^−1^ lysozyme, 4.5 g L^−1^ citric acid and 5 g L^−1^ chitosan that can be used to improve the formulation of marine *Arthrobacter oxydans* KQ11 supplemented dextranase mouthwash. Therefore, this study provided a foundation for the future design and development of oral care products using marine derived enzymes [36].

The dextranase from the psychrotolerant isolate *Catenovulum* sp. DP03 [78] found to at least partially prevent *S. mutans* from forming biofilms on glass coverslips. The dextranase was found to prevent extensive biofilm formation by *S. mutans* with a minimum biofilm reduction concentration (MBRC) MBRC_50_ of 20–40 U mL^−1^ [78]. Likewise, dextranase by *Arthrobacter oxydans* KQ11 exhibited an excellent effect to prevent dental plaque with an MBRC_50_ 5 U mL^−1^, MBIC_50_ (minimum biofilm inhibitory concentration) and MBIC90 were 2 and 6 U mL^−1^, respectively [81].

In a study carried out by Ning and coworkers [35], the effect of dextranase produced by the strain PX02 from *Cellulosimicrobium* sp. on preventing dental plaque biofilm was studied that exhibited the MBRC_50_ and MBRC_90_ as 9 and 15 U mL^−1^, respectively [35]. A novel dextranase (BaDex) from *Bacillus aquimaris* S5 could effectively remove 80% of dental plaque at 8 U mL^−1^ in MBRC experiment [84].

In addition to plaque removal, the dextranase enzyme has an important industrial application in medicine, where dextranases are used to partially hydrolyze native dextran in the production of blood substitutes. In addition, the enzyme is used to produce low molecular weight dextran and a cytotoxic dextran conjugate, and dextranase has been shown to enhance antibiotic activity in endocarditis. The use of dextranase in sugar factories not only improves productivity, but also the quality of the sugar, because polysaccharides interfere with the sugar production process, resulting in large losses for sugar factories; thus, the removal of dextran is required to improve the characteristics of sugar in sugar factories. Excessive crystal elongation and viscosity of syrups and molasses can also be reduced by enzymatic degradation of dextran [38].

## 6. Conclusions

The classification and characteristics of dextran hydrolyzing enzymes, as well as the production processes and functional characteristics of marine dextranases, were surveyed and discussed in this study based on the analysis of published literary sources. This review compared dextranases from marine bacteria with dextranases from other microorganisms. The review presented various sources of dextranase-producing microorganisms like bacteria and fungi isolated from various marine sources: shrimp, fish, seaweed, sea slit, and sea water. The effect of marine dextranase on plaque removal was also described in brief. The study has enabled to explore novel applications of marine bacterial dextranase in present and future course of research era. Since dextrans have enormous prebiotic properties, their effect on food texture and their reliability must be carefully evaluated. In addition, dextranase is used to produce low molecular weight dextran and a cytotoxic dextran conjugate, and it has been shown to enhance antibiotic activity in endocarditis.

Hence, the studies of marine bacterial dextranases for several use including oral hygiene products require more research and breakthrough technologies for their final application. 

## Figures and Tables

**Table 2:** The physicochemical properties of marine bacterial dextranases.

Species	Enzyme	Enzyme Family	Enzymatic Digestion Products	pH (Opt)	Temp (Opt)	Stable at pH	Stable at Temp	Activator	Inhibitor	Ref
*Arthrobacter* sp.	Dex410	Endoglycosidase	isomaltotriose, isomaltoteraose, and isomaltopentaose	5.5	45	-	-	-	-	[34]
*Cellulosimicrobium* sp. PX02	-	-	isomaltotriose, maltopentaose, and isomaltooligosaccharide	7.5	40	7.0–9.0	up to 45	-	-	[35]
*Catenovulum agarivorans* MNH15	-	α-1,6-glucosidic bonds	glucose, maltose, and maltoheptaose	8.0	40	5.0–9.0	30 °C	Sr^2+^	NH_4_^+^, Co^2+^, Cu^2+^, Li^+^	[76]
*Catenovulum* sp.	Cadex	α-1,6 glycosidic bond	Isomaltoogligosaccharides	8.0	40	5.0- 11.0	under 30 °C	Mn^2+^, Sr^2+^	Cu^2+^, Fe^3+^, Zn^2+^, Cd^2+^, Ni^2+^, Co^2+^	[77]
*Catenovulum* sp. DP03	-	-	-	8.0	40	6.0–11.0	30	-	-	[78]
*Catenovulum* sp. DP03	Cadex2870	-	maltose, maltotetraose, maltopentose, maltoheptaose and higher molecular weight maltooligosaccharides	8	45	60% activity at pH 5–9 for 1 h	10% catalytic activity at 0 °C	-	-	[79]
*Arthrobacter oxydans* KQ11-1	-	-	glucose, maltose, maltotriose, and maltotetraose	6.5	60	-	-	-	-	[81]
*Arthrobacter oxydans* KQ11	-	-	-	7.0	50	-	>60% activity at 60 °C for 1 h	Co^2+^, Ca^2+^, xylitol, alcohol	Ni^2+^, Fe^3+^, 0.05% SDS	[83]
*Bacillus aquimaris* S5	BaDex	66	-	6.0	40	-	80% after incubation at 10–30 °C for 3 h	-	-	[84]

**Table 3:** Recombinant production of dextranase from marine bacterial origin.

Bacterial Strain	Dextranase Gene	Primer	Host Cell	Vector Plasmid	GeneBank Accession Number	Ref
*Arthrobacter* sp.	*Dex410*	27F (5′-AGAGTTTGATCCTGGCTCAG-3′) and 1492R (5′-GGTTACCTTGTTACGACTT-3′)	*-*	-	JX481352	[34]
*Arthrobacter oxidans* KQ11	*Aodex*	-	*E. coli* DH5α and *E. coli* BL21 (DE3)	pCold III-KQ	KJ571608	[37]
*Catenovulum agarivorans* MNH15	*-*	27F (5′-AGAGTTTGATCCTGGCTCAG-3′) and 1492R (5′-GGTTACCTTGTTACGACTT-3′)	*E. coli*	pMD19-T	-	[76]
*Catenovulum* sp.	*Cadex*	-	*-*	pMD19-T	-	[77]
*Catenovulum* sp. DP03	*Cadex2870*	F (5′-GAAGATCTGGGCTGCTCAAGCAGCAGCTCGT-3′) and R (5′-ATAAGAATGCGGCCGCAATTTCGA TTTTTGTAATTTGATA-3′)	*E. coli* BL21(DE3)	pET29a	-	[79]
*Arthrobacter oxydans* KQ11	*DexKQ*	KQ-28aF: GGGAATTCCATATGAAGCATTACCTCCGTCTA; KQ-28aR: CCCAAGCTTCC-ACGCGTTCCAGTTATCCCA	*E. coli* BL21(DE3)	pET28a	AHZ97853.1	[80]
*Arthrobacter oxydans* KQ11-1	*-*	5′-CGCGGATCCCAGGAGCCCCGCTGCGACAGA-3′ (BamHI site is underlined) and (5′-CCCAAGCTTCCACGCGTTCCAGTTATCCA-3′ (HindIII site is underlined)	*E. coli* DH5α	pET-28a-(+)	D00834.1	[81]
*Arthrobacter* KQ11	-	5′-CGCGGATCCCAGGAGCCCCGCTGCGACAGA-3′ and 5′-CCCAAGCTTCCACGCGTTCCA TTATCCA-3′	*E. coli* DH 5α	PMD-19	KJ571608	[82]
*Bacillus aquimaris* S5	*BaDex*	F (5′-CGCGAGCTCATGGGGAAAAAGAA-3′) and R (5′-CCGCTCGAGTTTATAGTCGATCACGACC-3′)	*E. coli* BL21(DE3)	pET29a	-	[84]

**Table 4:** Comparative characteristics of sources for producing dextranase according to the results of the analysis of literature sources.

Dextranase Source	Advantages	Disadvantages
Mold fungi	high enzymatic activity	some mold fungi can be poisonous; fungal spores are volatile and can contaminate production facilities
Bacteria	fast cultivation; high enzymatic activity	development of a complex isolation method is required
Yeast	there is no bacterial DNA in the preparations produced by cultivating yeast	long cultivation period
